# Nutritional intelligence in the food system: Combining food, health, data and AI expertise

**DOI:** 10.1111/nbu.12729

**Published:** 2025-01-12

**Authors:** Danielle I. McCarthy

**Affiliations:** ^1^ Queen's University Belfast Belfast UK; ^2^ Spoon Guru London UK; ^3^ Nutrition Talent Bangor UK

**Keywords:** AI, evidence‐based practice, food industry, machine learning, nutrition, personalisation, quality assurance

## Abstract

Transformative change is needed across the food system to improve health and environmental outcomes. As food, nutrition, environmental and health data are generated beyond human scale, there is an opportunity for technological tools to support multifactorial, integrated, scalable approaches to address the complexities of dietary behaviour change. Responsible technology could act as a mechanistic conduit between research, policy, industry and society, enabling timely, informed decision making and action by all stakeholders across the food system. Domain expertise in food, nutrition and health should always be integrated into both the development and continuous deployment of AI‐powered nutritional intelligence (NI) to ensure it is responsible, accurate, safe, useable and effective. Dietary behaviours are complex and improving diet‐related health outcomes requires socio‐cultural‐demographic considerations within the design and deployment of NI tools. This article describes existing examples of NI within the food system and future opportunities. Human‐in‐the‐loop approaches with food, health and nutrition experts involved at all stages including data acquisition, processing, output validation and ongoing quality assurance are essential to ensure evidence‐based practice. The same ethical considerations should apply in this domain as in any other (e.g. privacy, inclusivity, robustness, transparency and accountability) and responsible practice must encompass rigorous standards and alignment with regulatory frameworks. Critical today and in the future is accessibility to appropriate high‐quality food compositional data sets, which include up‐to‐date information on commercially available products that reflect the constantly evolving food landscape to realise the potential of responsible AI to help address the existing food system challenges.

## BACKGROUND

There is an urgent need for a coordinated global effort to improve the quality of the human diet, which recognises that dietary behaviours are complex and improving diet‐related health outcomes will require active collaboration between stakeholders across the food system. Suboptimal diet is an important preventable risk factor for non‐communicable diseases. In a systematic, global analysis, 11 million deaths a year and 255 million disability‐adjusted life‐years (DALYS) were attributed to dietary factors (Afshin et al., 2019).

Current dietary behaviours also have substantial negative implications on planetary health, with an increasing amount of research investigating dietary optimisation approaches that are within planetary boundaries (Eustachio Colombo et al., 2024). It is pertinent that interventions at multiple levels across the food system are made that ensure planetary, as well as, public health, is improved and protected, and for healthy and sustainable diets to become the new normal (Dimbleby, 2021). However, translating this into globally applied practice is a colossal task.

In the UK, less than 0.1% of diets sampled from the *National Diet and Nutrition Survey* were found to adhere to all nine of the recommendations within the Eatwell Guide, the national food‐based dietary guideline in the UK, with less than a third (30.6%) adhering to at least five recommendations (Scheelbeek et al., 2020). This disconnect between healthier dietary guidance and food choice highlights a clear need for more support to enable behaviour change at scale. The *Food4Me* study found a personalised approach had bigger, sustained changes in eating behaviour after 6 months compared with standardised healthy eating advice (Celis‐Morales et al., 2017). In a follow‐up article it was highlighted that for such research to be converted into action that has a significant impact on public health, it would need to address the psychological, social, economic and cultural factors that are known to influence eating patterns (Mathers, 2019).

The latest wave of the UK Food Standards Agency's *Food and You 2* survey reported a 9% increase in food insecurity over the past 3 years with levels reaching 25%. Findings also showed that 80% of respondents reported that they had made changes to their eating habits for financial reasons in the past year (Armstrong et al., 2023). It is, therefore, critical that food and nutrition insecurity are considered within the system‐wide approaches needed to improve health. Existing health disparities must be actively addressed and equitable solutions created for individuals with and without food security (Brandt et al., 2023). Inclusivity and contextual appropriateness should be key success factors in the design of multifaceted whole‐system approaches to ensure solutions are individually appealing, appropriate, available, accessible and affordable for everyone. For example, the rights of those individuals and families affected by food allergy should be protected with access to safe, affordable, nutritious food, regardless of their food allergy (Jefferson et al., 2024). This should also extend to those with dietary preferences based on cultural beliefs, for example, those following Halal‐based food practices.

In a systematic review of the determinants of consumer acceptance, use and adherence to personalised dietary advice, the authors highlighted the potential of utilising machine learning (ML) in the delivery of dietary interventions to speed up the development of real time, relevant personalised nutrition (Reinders et al., 2023). Precision nutrition (PN) research aims to use personal information to deliver individualised nutrition advice rather than generic advice. The potential of ML in PN research to facilitate integration of multiple complex features to deliver high‐performance personalised approaches for treatment, prevention or maintenance of optimal health has been recognised (Kirk et al., 2021). A role for ML in the classification of food using pictures has also been identified as a potential complementary tool to help improve precision and validity within nutritional epidemiology (Morgenstern et al., 2021). Limitations noted in this review, and relevant to all artificial intelligence (AI) use cases, are that a model is only as good as the data it uses and that there remains a need for large‐scale trials to test the health impact of such approaches compared with population‐based approaches whilst also considering feasibility and cost–benefit (Kirk et al., 2021).

A review of existing digitally enabled tools noted the increasing use of AI models and their potential to revolutionise nutrition and health by making things easier for individuals and helping health professionals offer more personalised treatment (Abeltino et al., 2024). For the potential of AI to be realised within the food system, it is critical that the design and integration of such technology is done in ways that are continually responsible, evidence‐based, accurate, accessible, affordable and safe as well as effective. This requires an open, transparent and collective approach to the integration and evaluation of such technologies. The aim of the present article is to consider the design, deployment and potential limitations of AI‐based tools in the context of the food system with a focus on health and nutrition and the need for responsible and ethical practices. Insights of existing applied practice are shared from the perspective of the author who holds the position of Chief Health Officer at Spoon Guru, an AI‐nutrition technology company.

## DEMYSTIFYING AI

AI leverages computers and machinery to simulate decision making (Xu et al., 2021). ML is a subdivision of AI that describes a subset of algorithms that are designed to learn from the data with which they are presented and identify complex patterns whilst also being capable of collecting and processing large amounts of unstructured data that traditional data techniques could not (e.g. free text, images and audio) at a scale that is unmatched by humans (Kirk et al., 2021). Natural language processing (NLP) is the term used to describe the specific field of computer science used to interpret written and spoken text in a way that is understood by a computer.

A recent study that applied a NLP approach to encode texts from food labels and ML to automate the categorisation of foods and calculate nutrition quality scores reported high accuracy (>97%) of the pre‐trained language model (Hu et al., 2023). Authors concluded that such methods reduce the time needed for manual categorisation and nutrition quality score calculation and that such automation is effective, feasible and reliable and could provide timely analysis of the impact of food policy on food supply (Hu et al., 2023).

There are different types of language models, small and large. Large language models (LLM) offer a broad comprehension of language, exemplified by entities like Chat GPT, compared with small language models (SLM), which offer highly precise, context‐aware information that can drive accuracy and enable specialisation. For example, using NLP and ML, Spoon Guru has developed a food and nutrition‐based small language model producing a nutritional and dietary nomenclature and ontology that discerns between food groups, nutrients, ingredients, allergens, dietary and cultural preferences. In the context of nutrition, small language models can decipher complex dietary information and utilise large language models to enable comprehension of word relationships and sentence structures in ways that reflect the domain expertise (e.g. nutrition), upon which the small language model is defined to deliver informed, accurate nutrition counsel at scale. It is through the combination of these capabilities that responsibly trained SLMs can excel in identifying linguistic subtleties that their LLM counterparts may inadvertently overlook. The key advantage of SLMs is that they bring grounding to the approach, integrate specific domain expertise, domain‐specific language, performance, updatability and interoperability.

Utilisation of responsibly sourced data and integration of domain expertise within verifiably trained SLMs is being used to detect ingredients, map these to food groups, integrate dietary guidance principles, whilst also identifying suitability with various dietary conditions (e.g. presence of specific allergens) (Spoon Guru, 2024a). Such approaches have been applied at scale globally within food retail. They are highly adaptable for deployment across the food system with the capability to evolve at pace in ways that previously human capacity could not facilitate. This tech‐enabled functionality represents a novel conduit within food system processes that enables the integration and continuous application of criteria at scale to existing food information (e.g. nutrition composition, ingredients and processing procedures). This represents an opportunity to understand at scale alignment of practices with dietary guidelines, health policy and legislation and to provide dietary and food expertise at scale and in context across the food system.

Generative AI (gen AI) is a domain of AI that takes various inputs (e.g. from video, text, images, audio) and generates new content (Lv, 2023). An example of its utilisation in a food context is in recipe development where it can be used to generate new recipes based on preferences and specific ingredients or lunch suggestions with available ingredients such as Meal Reveal (Unilever, 2024). Such technology could be utilised to generate individualised meal plans that meet taste and cultural preferences whilst fulfilling nutritional needs, dietary and lifestyle requirements in ways that are contextually relevant (e.g. location, social setting, time availability, capability and affordability). The utilisation of SLMs within gen AI solutions could deliver highly precise responses to nutrition‐related queries (e.g. that align with the dietary guidance and scientific evidence upon which the model was trained).

This technology, also known as conversational AI, represents a route to innovation that can help users engage verbally with information. Virtual assistants, if underpinned by models that have been trained responsibly and based upon appropriate data, could deliver responses based on an understanding of the multitude of complex needs that define each individual's food requirements, with the adaptability to alter outputs as needs change, within and/or between days and contexts. As scientific understanding and data availability evolve, real‐time dietary counsel at scale could consider multiparameter data (e.g. metabolic markers, health conditions, genotype, phenotype, microbiome, nutrient and non‐nutrient components, sustainability components, food production and processing records, behavioural and demographic data and wearable outputs). The possibilities are endless. It will be critical that such approaches are designed, deployed and evaluated robustly, with appropriate subject matter expertise. It will also be essential to ensure that data privacy and ethical considerations are central to the developments in this area. A combination of transdisciplinary expertise and cross‐sector experience will be fundamental to progress in this field and ensure what could be perceived as great promise delivers meaningful impact.

Simplification of the journey for everyone towards healthier sustainable choices that are appealing, simple to make, easily accessible and affordable should arguably be the central target for the transformational behaviour change required to improve public and planetary health. It could be imagined how technology, if available to everyone, could stand as an important mechanism in addressing health inequalities and solutions for all. Indeed, in the future, with developments in Gen AI it is possible that many would have an opportunity to voice their needs and create demand based on their specific requirements and contexts. SLMs could be utilised to identify patterns within such complex data sets and generate insights which could inform food businesses, health professionals and civic leaders to restructure and offer propositions that meet the complex needs of those they serve. Verified and responsibility‐created SLMs underpinning such innovations are essential. Efforts must actively address existing inequities and discrimination to ensure digital developments do not accelerate these (Rosales & Fernández‐Ardèvol, 2020).

## AI‐BASED NUTRITIONAL INTELLIGENCE: APPLIED EXAMPLES

In food retail, NLP approaches like those evaluated by Hu et al. (2023) are currently being applied at scale to categorise food and drinks based on nutritional composition, ingredients, the presence or absence of allergens and fit with cultural, lifestyle or dietary requirements. Whilst straightforward dietary‐based data filters (e.g. low sugar, low salt) are based on simple criteria that can be determined from nutrition label information, composite health, dietary and cultural filters need to be created using much more complex criteria. Both require carefully considered expert‐informed processes to ensure appropriate, accurate outputs that are usable and useful.

Spoon Guru is an example of a technology company, specialising in nutritional intelligence (NI) that has been developing and deploying such approaches at scale to enable global food retailers to categorise their product ranges using deep food and health‐based criteria and expertise for nearly a decade (Spoon Guru, 2024b). A human‐in‐the‐loop (HITL) approach is taken to deliver advanced machine learning algorithms that enable complex dietary label attribution that identify and signpost products that meet multiple criteria (e.g. category based) and/or scoring approaches based on different nutrients and/or product constituents. A HITL approach to ML is based on interactions between humans and ML algorithms at various points in the ML process (Mosqueira‐Rey et al., 2023). In Spoon Guru, this approach involves both technical experts who manage data cleansing and optimisation of product data and nutrition experts who ensure data appropriateness and quality and develop complex rules to ensure label attributions are reflective of scientific evidence and in‐market regulations. In the context of the food system, the involvement of food and nutrition experts throughout the process, which extends beyond attribute design and includes deployment, is essential to ensure ongoing quality, accuracy and utility of outputs in a continually changing food environment. As food nomenclature, product ranges, formulations and recipe repositories change all the time, continuous expert‐informed quality assurance processes are critical to the robust delivery of NI. Evaluation of the utility and impact of label attributions in practice is also important to inform responsible and effective use of NI. Social, behavioural and implementation expertise can also be integrated to ensure usability and usefulness of outputs. This multi‐stranded tech‐enabled approach to evidence‐based health signposting at scale, which combines research evidence, practitioner expertise and individual requirements are key considerations to help influence uptake, adherence and effectiveness of the digitally enabled activities the label attribution outputs enable (Hickson et al., 2024).

Utilising technology underpinned by responsible methodology such as that described above has a number of potential benefits: (i) simplicity, scalability, feasibility; (ii) consistency; and (iii) adaptability.

### Simplicity, scalable, feasibility and utility

Utilising responsible NI on product data can surface a non‐exhaustive amount of label attributes on an unlimited number of products which can integrate simple to complex criteria, for example, nutrient and/or food group composition, ingredients, dietary or lifestyle requirements and/or health benefits, and process this to deliver simplified labels that act as signposts that can be individually contextualised and delivered in real time. For context of scale, Spoon Guru to date has processed data on 1.2B products and 75 000 ingredients. This level of data processing and accurate label attribution on a product‐by‐product basis would not be feasible without using NI. Automated attribution of digitalised signposts at such scale and pace increases the ‘discoverability’ of products with the attribute of interest in real time. Maximised, accurate coverage is critical to feasibility and utility in practice.

Robust tech‐enabled label attribution can be utilised in a number of ways within the food retail setting, for example, to bring a health focus to incentive programmes delivering targeted vouchers and discounts; to deliver health‐orientated paths to purchase (e.g. displays online or instore), inform marketing strategies and product placement. Research has shown when a combination of these approaches is delivered within a food retail setting there are benefits with respect to health outcomes and cost‐effectiveness (Yoder et al., 2021). Label attributions can also be used to inform product swap recommendations at scale which align with individual shopper's goals and contexts. Other data can also be integrated as part of label attribution (e.g. price). The digitalisation of nutrition and health‐based attributes at scale holds great potential in enabling individuals to access, and indeed, create, food environments that best reflect their needs.

In the US, the Spoon Guru NI technology was used to convert complex nutrition, food group and ingredient‐based criteria into a simple ‘Dietitian's Pick’ signpost, which was integrated into a retailer's healthier habits loyalty programme. Analysis shows encouraging early results (Spoon Guru, 2024b).

Scalability can be impacted by the availability and accessibility to the technology. Platform partnerships can represent one way to increase the potential to deliver tech‐based solutions at scale. For example, the Spoon Guru NI technology is available on the Google Cloud Marketplace, which can help with the integration of the technology at scale.

### Consistency

There has been a call made by some stakeholders within the food system for more of a level playing field across food businesses when it comes to action on obesity (House of Lords, 2024). Recommendations were made In the National Food Strategy for all food companies, including retailers, restaurants, quick service companies, contract caterers, wholesalers, manufacturers and online ordering to publish consistent health metrics via an online portal (Dimbleby, 2021). Currently, the UK Food Data Transparency Partnership, which aims to improve the availability, quality and comparability of data in the food supply chain, is considering such metrics. The latest meeting report noted the need for consistency in reporting against clear methodology. Feasibility for businesses was noted as a key factor to balance against this need for consistency. Food data availability, quality, completeness, linkage to sales data and the resource and time intensity involved were all noted as key issues that are difficult to fix (Food Data Transparency Partnership Health Working Group, 2024). Responsibly designed NI is well‐paced to address each of these issues.

Another issue highlighted in this report was the use of inconsistent proxies when data are missing. For example, when calculating the percentage of fruit, vegetables, nuts (%FVN), a component of the UK Nutrient Profiling Model (NPM), this information is not available on pack. As a result, there is variability in approaches taken to address this challenge (e.g. use of zero values or average amounts per product category) (Food Data Transparency Partnership Health Working Group, 2024; Jenneson et al., 2020). Such methodological variations may impact the quality and utility of subsequent categorisations (e.g. high fat, sugar, salt foods [HFSS] and non‐HFSS products). Methodological variability in addressing this FVN data challenge may also impact the ability to measure and compare changes in FVN levels over time, an important component of healthier reformulation programmes. NI is well placed to solve such issues and enable standardisation in the translation of such profiling systems into practice across the food system. A NI‐based proprietary FVN analyser has been designed and deployed by Spoon Guru which uses NLP to make sense of the ingredient list and apply appropriate food groups classification to consistently calculate a %FVN calculation on a product‐by‐product basis. The tool can be utilised for packaged, unpackaged products and recipe data and thus holds significant potential to increase accessibility to a consistent, efficient approach to complex nutrition quality scoring and associated categorisations across the food system.

NI could also help deliver an empowerment tool for those SMEs or businesses in the out‐of‐home sector that currently experience barriers to participation in health policy‐related activities (e.g., a lack of data availability, capacity and limited access to nutrition expertise). NI approaches such as those described in Table 1 are currently delivered for recipes as well as food products. Thus, this technology could be utilised to provide a standardised, quality‐assured approach at scale that is consistent across the food system. In the same way, it is utilised by shoppers in food retail today, it could be utilised by others with significant agency in the food system, such as those responsible for public food procurement. Accessibility to NI‐enabled tools could ensure all those that make food‐based decisions have access to evidence‐based, informative, insights and recommendations that could help support healthier decision making.

**TABLE 1:** Summary of a number of potential use cases for nutritional intelligence (NI) across the food and healthcare system.

Food and healthcare context	Potential use cases for responsible NI
Food service	Customer decision making; menu creation; health metric reporting
Dietary advice/support	NI could be used to apply dietitian defined criteria to food products or recipes that align with condition management advice enabling dietary advice at point of food purchase and/or preparation that aligns with clinical need NI could be used to design and deploy food prescriptions and enable the conduit between health professionals, food provision and individuals
Policy creation/implementation	NI could enable scenario planning at speed using customised criteria to identify products by nutrient/ingredient criteria. This could help inform food and health policy creation by enabling efficient sight at scale of impacted products and also enable implementation planning by automatically identifying all products/recipes impacted with indication of areas of adaption required
Health metrics	Health reporting using standardised NI enabled methodology could be used to apply consistent and complex nutrient‐/food group‐/ingredient‐based criteria across all products in the food sector presenting a consistent, efficient, accessible enabler for all stakeholders to participate in health reporting
Research at scale	Large‐scale dietary interventions could be delivered at scale utilising NI to integrate dietary methodology into label attribution enabling signposting in a variety of contexts for participants (e.g. recipes, food shop, food service environments) NI that integrates conversational AI could enable novel citizen science dietary research at scale
Special diets	Menus (e.g. across schools) could be designed efficiently and safely utilising responsible NI to generate suitable recipes and ingredient swaps
Food Procurement	NI could enable healthier food procurement at scale. NI informed signposting would enable efficient, informed selections to be made of food products and meals that are of better nutritional quality within certain parameters (e.g. price). NI could be used to identify at scale the compliance of food products or menu items that comply with published standards (e.g. School Food Standards).

Abbreviation: AI, artificial intelligence; NI, nutritional intelligence.

### Adaptability

Definitions of healthy, sustainable diets are evolving and will continue to evolve to include multiple components beyond the traditional nutrients included in existing models (energy, fat, saturated fat, salt, sugar, protein, fibre, fruit, vegetables and nuts). For example, the food compass which considers additional nutrients, non‐nutrients, processing aids (Mozaffarian et al., 2021), or the developing blended profiling systems that consider environmental components as well as nutritional (Grigoriadis et al., 2021). NI that can adapt at pace to new definitions could be critical to speeding up the translation of research into implementation and impact at scale in ways that also can integrate socio‐cultural and demographic needs. As research into precision nutrition develops, such as that explored in the *PREDICT* studies (Berry et al., 2020), and areas for increased efficiency are confirmed, NI technologies could be utilised to help integrate recommendations across the food system and help enable accessibility to all.

Other applied examples of AI‐based tools within the food system highlight the diversity of technology‐enabled activity. For example, Walmart launched a generative AI search in January 2024 to help customers find their desired products quickly, saving time. Utilisation of technology in this most recent development enables customers to utilise the search function using natural language prompts based on their need or context, such as ‘hosting a party’ rather than conducting a series of individual searches based on different product categories (e.g. party supplies, savoury snacks) (Anon, 2024b). Instacart has launched an AI‐enabled ‘Ask Instacart’ which allows customers to search based on themes of interest (e.g. dinner or date night rather than by item) (Zhuang, 2023). Amazon has utilised AI over the past 25 years to improve customer experience and deliver propositions such as drone delivery, checkout‐free stores and the conversational Alexa (Mehta & Chilimbi, 2024). Most recently, AI shopping assistant Rufus, a gen AI‐powered expert (shopping assistant trained on information from customer reviews, product catalogue and information on shopping needs and products) has been launched to deliver recommendations in a conversational context (Mehta & Chilimbi, 2024). A cohort of companies are utilising AI to tackle food insecurity by optimising distribution, for example, San Francisco‐based Replate redirects surplus food to nonprofits, taking into consideration the types of food—such as its nutritional value and whether it is suitable for specific dietary needs—as well as the demographics of its recipients (Anon, 2024a; Bernabe, 2022).

## AI‐POWERED NUTRITIONAL INTELLIGENCE: RESEARCH EXAMPLES

From a nutrition research perspective, the authors of a systematic review noted a great and growing interest in the use of machine learning algorithms to evaluate food consumption and recommended such studies be conducted in each country to ensure problems faced in different regions are identified (Oliveira Chaves et al., 2023). Martin‐Morales et al. (2023) highlighted the utility of dietary intake in machine learning‐based data analysis in their investigations of cardiovascular mortality. Kirk et al. (2022) in a paper designed to provide a guide to ML for nutrition researchers described case studies on precision nutrition and metabolomics, identifying these as research domains for which ML is highly applicable. Berry et al. (2020) utilised ML to consider PN and deepen understanding of meal composition, habitual diet, meal context, anthropometry, genetics, microbiome, clinical and biochemical parameters and how these predict triglyceride and glycaemic response to food intake.

ML techniques were utilised recently to explore genetic markers from metabolite and genome‐wide association studies to predict the multi‐nutritional properties of pigmented rice, demonstrating how integration of such technologies within research methodology can help generate new knowledge at pace (Tiozon et al., 2023). Guess (2024) recently reflected on the algorithms that can capture medical, nutrition, wearable and gut microbiome data to enable dietary recommendations that are tailored to an individual's need and drew attention to the research questions that still need to be answered to determine if personalised nutrition and utilisation of big data (biological, behavioural, social and environmental) makes more precise, effective, individualised dietary recommendations that have greater clinical utility compared with generic dietary advice. As technology enables new research questions to be explored in ways previously unavailable, efficacy will be an essential consideration within implementation practices.

## FUTURE OPPORTUNITIES

Technological advancements present a potential enabler of multifactorial, integrated approaches that can be delivered to help empower informed, effective transformational decision making across the food system. A collaborative approach between AI models and humans can help drive efficiency and scale (Mosqueira‐Rey et al., 2023).

Extending utilisation of NI beyond food retail to enable scalable attribution of robust health signposting could be helpful in many other contexts in the food and healthcare system. This paper has focused largely on the utilisation of AI in the context of food product data, In future the integration of AI with individual‐level consumption and wider behaviour data represents a significant area for further exploration.

## KEY CONSIDERATIONS AND RECOMMENDATIONS

An absolutely critical need today and in the future is accessibility to high‐quality food compositional data sets that include up‐to‐date branded and generic food information that reflects the constantly evolving food landscape. Currently, branded food data sets can originate from a variety of sources, alongside national food composition data sets, but these are fragmented, and data are not readily found, accessible, interoperable or reusable (FAIR) (Pravst et al., 2021). This has been addressed from a research perspective in the FNS‐Cloud project and recommendations have been made for data managers in the research setting to produce interoperable open data (Emara et al., 2022). These recommendations are also highly relevant and essential for the robust translation of research into practice, with standardisation and data accessibility critical to success (Eftimov et al., 2017). The current lack of open accessibility to high‐quality appropriate food compositional data, which includes up‐to‐date information on commercially available food products, is a limitation to realising the potential of responsible NI to help address the existing food system challenges.

To realise the potential of responsible NI, tools must be designed and deployed in ways that deliver utility, feasibility, scalability and proven efficacy. Domain expertise in food, nutrition and health should always be integrated into both the development and continuous deployment of NI. These experts must play significant roles at all stages including data acquisition and cleansing, processing, output validation, whilst also contributing to ongoing quality assurance processes to ensure appropriate, evidence‐based, accurate outcomes in an ever‐changing environment. In addition, the personal and professional lived experiences of stakeholders across the food system should be integrated to ensure the technological solutions developed to solve the complex challenges faced are done so in ways that are inclusive, contextually relevant, efficient, usable and progressively effective.

To realise the required transitions in behaviours that are defined by food, nutrition, health and sustainability expertise, complex socio‐cultural‐demographic navigation is also required and should be considered in the design and deployment of responsible NI. For example, gender differences have been reported that suggest that women are more disposed to a sustainable diet compared with men (Henchion et al., 2022). The same ethical considerations should apply in this domain as in any other within the food and healthcare systems (e.g. privacy, inclusivity, robustness, transparency and accountability). Responsible practice must encompass rigorous standards and align with existing regulatory frameworks (e.g. GDPR, Human Tissue Act, food legislation, consumer and data protection). In recognition of the critical importance of ethical approaches to, and in the utilisation of, AI, initiatives such as the ‘Me‐We‐It: An Open Standard for Responsible AI’ initiated by the World Ethical Data Foundation have been established (World Ethical Data Foundation, 2023). Such endeavours are designed to ensure AI applications adhere to rigorous ethical benchmarks. As transformational technologies are integrated in different ways across the food system, it is essential that informed frameworks are published to protect citizens, address bias and ensure issues such as copyright are dealt with effectively.

To leverage robust technological solutions to both explore and implement scalable evidence‐based solutions, efforts are needed to address the complexities that impact trust in AI‐based tools. Research has highlighted that ethical and regulatory frameworks, policies and partnerships and evaluations that identify reliable solutions are needed (Rejeb et al., 2022). Technological transparency, open, problem‐solving mindsets, alongside fast‐paced, targeted, test, evaluate and learn approaches are needed for the potential of robust technology‐based solutions to assist experts in the food and health system to tackle the challenges they face.

There are a number of large, multi‐centre, transdisciplinary research studies underway that demonstrate connectivity and a collaborative approach between a wide variety of domain experts and those with lived experience of challenges faced. For example, the Food Insecurity and People Living with Obesity (FIO Food) project brings together behavioural scientists, psychologists, data scientists, clinicians, those with nutrition and food insecurity expertise and those with lived experience of the key food and health issues many in our society face (e.g. patients, food system stakeholders, individuals and policymakers) (Lonnie et al., 2023). A white paper was recently published by this group that explored stakeholders' experiences and described a framework for action for policy and healthcare practitioners (Lonnie et al., 2023). The role that responsible NI could play in the implementation of such recommendations is worthy of consideration. Such approaches could work as a mechanism to enable large‐scale, free‐living interventions that are accessible to everyone, enabling participation by multiple food businesses spanning a number of food environments (e.g. food service, out‐of‐home, food retail, food delivery, high street).

The future is only going to get more complex with scientific developments in nutrition and sustainability and the ever‐changing world of technological developments. Approaches need to be continually connected and evaluated to ensure progress is based upon timely access to an evaluative evidence base. It is critical that technological developments should be used to actively address existing diet‐related health inequalities and certainly not extend these. There must be conscious consideration of potential unintended consequences. Data sets should be broad and diverse to ensure they are representative, and the methodologies upon which that data is processed and outcomes communicated should actively consider addressing potential bias.

To ensure inclusivity in the NI field, support (e.g. education and training pathways and collaboration opportunities) should be available for those not yet familiar with the technology in this context (Morgenstern et al., 2021). This will open opportunities for all stakeholders to become involved in technologically linked opportunities to help positively transform our food system. Explainable AI is a key element in the delivery of transparency and traceability in the field of NI, enabling end users in receipt of AI‐generated conclusions to understand how models have delivered their output (Kirk et al., 2021).

As food and health data are generated beyond human scale, there is an opportunity for humans and AI to collaborate to enable informed decisions and action by all stakeholders within the food system. NI tools that help navigate the complexities of food, public and planetary health and empower individual agency in ways that respect socio‐cultural factors have the potential to help drive the transformational changes required for public and planetary health.

## CONFLICT OF INTEREST STATEMENT

Danielle McCarthy is Chief Health Officer at Spoon Guru. The content of this article reflects her applied expertise working as a nutritionist in this role as well as other previous roles in academia, food manufacture, food retail and pharma.

## Data Availability

Data sharing not applicable—no new data generated, or the article describes entirely theoretical research.
